# Attitudes towards colorectal cancer screening in Lynch syndrome families: how do they change from pre-test genetic counseling to 6 and 12-months post-disclosure?

**DOI:** 10.1186/1897-4287-9-S1-P5

**Published:** 2011-03-10

**Authors:** Allison M Burton, Susan K Peterson, Salma K Marani, Sally W Vernon, Christopher I Amos, Marsha L Frazier, Patrick M Lynch, Ellen R Gritz

**Affiliations:** 1 Department of Behavioral Science, The University of Texas MD Anderson Cancer Center, Houston, TX, USA; 2 Division of Health Promotion and Behavioral Sciences, The University of Texas-Houston School of Public Health, Houston, TX, USA; 3 Department of Epidemiology, The University of Texas MD Anderson Cancer Center, Houston, TX, USA; 4 Department of Gastroenterology, Hepatology & Nutrition, The University of Texas MD Anderson Cancer Center, Houston, TX, USA

## Background

Adherence to colonoscopy recommendations can reduce colorectal cancer (CRC) morbidity and mortality. However, little is known about the use of colonoscopy among persons at risk for Lynch syndrome prior to clinical genetic testing (CGT) and how notification of mutation carrier status affects screening behaviors. The goal of this study was to evaluate colonoscopy use and attitudes towards CRC screening among unaffected relatives of Lynch syndrome mutation carriers before pre-test genetic counseling (baseline) and 6 and 12 months after disclosure of the test results. The primary outcomes of interest were colonoscopy utilization, commitment to screening for colonoscopy, self-efficacy regarding screening adherence, and the benefits, barriers, and efficacy of CRC screening.

## Methods

110 unaffected relatives completed a psychosocial questionnaire prior to pre-test counseling, 81 had GCT and received test results, and 78 subsequently completed follow-up questionnaires at 6 and 12 months post-results disclosure (52 were mutation negative and 26 were mutation positive). Linear mixed models were used to evaluate group comparisons in change over time. In addition to the main effects of mutation status and time, the time by mutation interactions were included as fixed effects in the model to test for differences in average rate of change over time between the two mutation groups.

## Results

**Figure 1:**
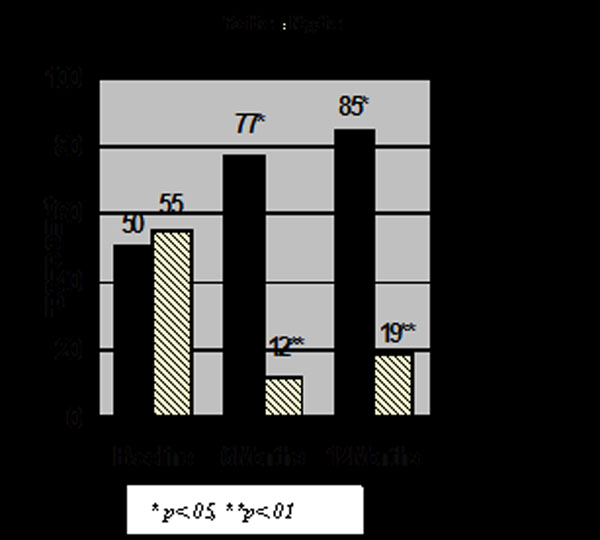
Percent Colonoscopy at Baseline and 6 and 12 Months after Disclosure of Lynch Syndrome Genetic Test Results

While both groups were similar at baseline, after disclosure of results, a significantly greater number of those with a positive test result had a colonoscopy compared with persons who tested negative where colonoscopy utilization decreased significantly (Figure 1).

Analysis showed a significant positive movement through the stages of readiness to have colonoscopy in the mutation-positive group from baseline to 6 and 12 month post-disclosure compared to a significant negative movement through the stages of readiness in the mutation negative group (Table 1). Analysis showed a significant increase in self-efficacy and benefits of CRC screening in the mutation-positive group from baseline to 6 and 12 month post-disclosure and no difference in the mutation-negative group. There was a significant decrease in barriers to CRC screening in the mutation-positive group from baseline to 6 and 12 month post-disclosure and no difference in the mutation-negative group (Table 1).

**Table 1:** Comparison of psychosocial measures by test result at baseline, 6 months post-results disclosure, and 12 months post-results disclosure (n=78)

	Positive (n=26)			Negative (n=52)		
	Baseline M (SD)	6 months M (SD)	12 months M (SD)	Baseline M (SD)	6 months M (SD)	12 months M(SD)

Readiness (% committed)	58%	81%*	88%*	69%	51%	62%
Self-efficacy	85% (28)	96% (8)*	96% (11)*	89% (22)	90% (19)	87% (25)
Benefits	4.4 (0.6)	4.6 (0.5)*	4.8 (0.4)*	4.4 (0.6)	4.5 (0.5)	4.5 (0.6)
Barriers	2.7 (0.6)	2.3 (0.7)*	2.7 (0.6)*	2.3 (0.7)*	2.3 (0.7)	2.2 (0.7)
Efficacy	4.1 (0.4)	4.3 (0.5)	4.3 (0.5)	4.1 (0.7)	4.1 (0.6)	4.1 (0.6)

* p<.05

## Conclusion

Our results show that CGT may motivate mutation carriers to have a colonoscopy after receiving test results and may also have an impact on their longer-term screening behaviors. In addition, CGT may have a positive impact on mutation carriers’ attitudes towards CRC screening.

